# Are nonintestinal inflammatory diseases and celiac disease linked? Nonintestinal inflammatory and celiac disease

**Published:** 2011-01-01

**Authors:** Roberto Bergamaschi

**Affiliations:** 1 Neurological Institute Mondino, Interdepartment Research Center for Multiple Sclerosis, Pavia, Italy

**Keywords:** Celiac, Virus Hepatitis

Dear Editor,

In their retrospective study, Leonardi and La Rosa (2010) did not find any instances of celiac disease (CD) among hepatitis B virus carrier patients [1]. CD is an inflammatory, immune-mediated intestinal disorder triggered by ingested wheat gluten in genetically susceptible individuals [2]. Leonardi and La Rosa (2010) found CD antibodies (immunoglobulin A and G antigliadin) in 11.6% of the cases, but no patient had immunoglobulin A antiendomysium or immunoglobulin A antitissue transglutaminase; consequently, a diagnosis of CD was ruled out. Even if a relationship between CD and hepatitis B virus had been present, the study could have been unable to detect a significant association due to the small sample size. Several studies have demonstrated a close association between CD and autoimmune disorders, such as insulin-dependent diabetes mellitus, thyroid disorders, dermatitis herpetiformis, Addison's disease, autoimmune myocarditis, Sjögren's syndrome, autoimmune hepatitis, autoimmune cholangitis, vasculitis, polydermatomyositis, systemic lupus erythematosus, and rheumatoid arthritis [3][4]. In the field of the neurological diseases, we reported a possible link between CD and optic neuromyelitis [5], an inflammatory disorder of the central nervous system of autoimmune etiology [6]. It is still debatable whether organ-specific autoantibodies sustaining CD represent an epiphenomenon or the expression of associated autoimmune disorders, but it seems very likely that an autoimmune environment might also trigger an abnormal response to gliadin. In the same way, hepatitis B and hepatitis C, which may have aminoacid sequences homologous to the toxic epitopes in gliadin, could trigger immunologic gluten intolerance in susceptible patients [7]. Thus, the hypothesis that nonintestinal diseases (such as hepatitis B and C or other inflammatory pathologies) may trigger immunologic gluten intolerance in susceptible subjects is still open to evaluation. Another controversial point investigated by Leonardi and Rosa [1] was the possible activation of CD due to the treatment of viral hepatitis with interferon-α (IFN-α; [8]) because of its immune modulating properties (e.g., producing dendritic cells that contribute to the Th1 response; [9]). Similar to CD, multiple sclerosis (MS), which is the most important neurological inflammatory autoimmune disease, is considered a "Th1 disease". Treatments of MS largely use a kind of IFN different from that used to treat hepatitis. Particularly, IFN-ß is able is able to reduce the risk of MS relapses through the down-regulation of proinflammatory Th1 responses [10]. In fact, no case of CD has been described among the numerous MS patients treated with IFN-ß in the literature so far. In regard to the different (and somehow opposite) mechanisms of IFN-ß compared to those of IFN-ß, the absence of CD activation in patients treated with IFN-ß could indirectly support the possibility of CD onset as a consequence of IFN-α therapy.
